# Resolutions of the New York Academy of Medicine

**Published:** 1871-11

**Authors:** 


### Resolutions of the New York Academy of Medicine

At a meeting held on the 21st of September, 1871, the following preamble and resolutions were unanimously adopted:—

*Whereas*, In charging the grand jury on September 6th (inst.), Judge Gunning S. Bedford said: "Of late, we have been living in an atmosphere of abortion. The very air is indeed heavy with the dark deeds of those heartless and unscrupulous specimens of human depravity, professional abortionists. Let the warning word this day go forth, and may it be scattered broadcast through the land, that from this hour the authorities, one and all, are to put forth every effort, and strain every nerve, until these traffickers in human be exterminated, and driven from existence, by fully vindicating the majesty of the law in all of these cases of its fiendish violation. Let me express the earnest hope—shared in, as I feel confident it will be, by you and by all right minded citizens—that the legislature at its next session will amend the statute-book, so that instead of reading "any person who shall administer to any woman with child, or prescribe for any such woman, or advise or procure her to take any medicine, drug, substance, or thing whatever, or shall use or employ any instrument or other means whatever, with intent thereby to procure the miscarriage of any such woman, unless the same shall have been necessary to preserve her life, shall, in case of the death of such child or of such woman, thereby produced, be deemed guilty of manslaughter in the second degree," it may read, "*shall be deemed guilty of murder in the first degree.*" Then the punishment would be death. Now, the crime being simply manslaughter in the second degree, the punishment is only imprisonment, not exceeding seven years:"

*Resolved*, That, in the opinion of the New York Academy of Medicine, the author of that language has, by so public a declaration of his sentiments, his intentions and his hopes, given us reason for renewed expression of highest commendation, has vindicated the already widely-expressed support from the medical profession of the country, of the course he has hitherto pursued, and has, we trust, greatly strengthened the esteem and confidence in which he is held by the public.

*Resolved,* That this Academy, in the discharge of the duty its professed objects commend—to promote public health and public morals—pledge all its influence and efforts, in support of any legislative or other measures which our law-officers may propose, as offering a reasonable promise of mitigating, if not removing, the pestilence of criminal abortion which is upon our country.

*Resolved,* That to remove all doubt from the public mind, in regard to the position of the New York Academy of Medicine in this important matter, to secure the influence upon the State authorities desired by this expression, and to stimulate the medical profession generally to similar acts, a copy of this preamble and these resolutions be forwarded to Judge Bedford, to District Attorney Garvin, and to the New York Bar Association; that the leading daily papers of this city, and its medical journals, be furnished with the same; and that the secular and medical papers and journals throughout the country be requested to copy.—*N. Y. Medical Journal.*

